# Correction to “Screening Mixed-Metal Sn_2_M(III)Ch_2_X_3_ Chalcohalides for Photovoltaic Applications”

**DOI:** 10.1021/acs.chemmater.3c02565

**Published:** 2023-11-01

**Authors:** Pascal Henkel, Jingrui Li, G. Krishnamurthy Grandhi, Paola Vivo, Patrick Rinke

The digital object identifier (DOI) that refers to all the calculation data we made publicly available on the Novel Materials Discovery (NOMAD) platform was misprinted. The correct DOI in sec. 2.1. “DFT Calculations” is 10.17172/NOMAD/2023.08.24-1.

